# Effect of Residual Pain After Posterior Fusion Surgery for Lumbar Degenerative Disorders on Health-Related Quality of Life: A Two-Year Follow-Up Using Patient-Reported Outcome Measures

**DOI:** 10.7759/cureus.61611

**Published:** 2024-06-03

**Authors:** Tatsuya Endo, Naohiko Kanemura, Toshikazu Ito, Keita Sato, Takuya Miura, Katsuya Onitsuka, Taku Miyazawa, Keisuke Kubota, Masumi Iwabuchi, Osamu Shirado

**Affiliations:** 1 Department of Orthopaedic and Spinal Surgery, and Rehabilitation, Aizu Medical Center, Fukushima Medical University, Aizuwakamatsu, JPN; 2 Department of Physical Therapy, School of Health and Social Services, Saitama Prefectural University, Koshigaya, JPN; 3 Department of Physical Therapy, Hokkaido Chitose College of Rehabilitation, Chitose, JPN; 4 Department of Physical Therapy, Tohto University, Fukaya, JPN

**Keywords:** heath-related quality of life, patient-reported outcome, posterior lumbar fusion, lumbar degenerative disease, residual pain

## Abstract

Study design: This is a prospective cohort study.

Purpose: The present study aimed to investigate the effects of residual pain after fusion surgery for lumbar degenerative diseases on quality of life (QOL).

Overview of literature: Residual symptoms after spinal surgery often restrict patients' activities of daily living and reduce their QOL. However, few studies have comprehensively addressed physical, psychological, and social factors.

Methods: The study population included a cohort of 208 patients (mean age: 67.9 years) who had undergone posterior interbody fusion for lumbar degenerative disease between 2012 and 2019. We asked the patients to complete the Japanese Orthopaedic Association Back Pain Evaluation Questionnaire (JOABPEQ) and Short Form Health Survey (SF-36) preoperatively, as well as at six, 12, and 24 months postoperatively. The presence of residual postoperative pain (RPP) was determined using the low back pain score of the JOABPEQ at six months postoperatively, and patients with an improvement of < 20 points compared to preoperative assessment were classified as RPP+ based on a previous study.

Results: In all patients, there was a notable postoperative improvement in all JOABPEQ and SF-36 domains compared to preoperative scores. The RPP+ group comprised 60 patients (69.6 years), while the RPP- group comprised 148 patients (67.2 years). In the RPP+ group, the lumbar function in the JOABPEQ and general health in the SF-36 showed limited postoperative enhancement. The pace of improvement in the role-emotional, role-physical, social functioning, vitality, and mental health scores was slower in the RPP+ group compared to the RPP- group.

Conclusions: In the current study, we found that the presence of residual pain at six months postoperatively affected QOL improvement up to 24 months after surgery. Lingering postoperative pain substantially impacted functional incapacity, social engagement, and psychological well-being. Notably, the lumbar function in the JOABPEQ and general health in the SF-36 showed distinct progression patterns in the RPP+ group.

## Introduction

Lumbar degenerative diseases, which include lumbar spinal stenosis, are age-related diseases, with an estimated number of more than 38 million patients in Japan [1]. Surgical treatment is indicated when symptoms are severe or conservative treatment is ineffective. However, symptoms associated with lumbar degenerative diseases may not completely resolve after lumbar fusion. Specifically, residual postoperative pain (RPP) left behind after surgeries often interferes with patients' activities of daily living and reduces their quality of life (QOL) [2]. Residual postoperative symptoms are reported to occur, with a frequency of 10-40% after lumbar spine surgery [3,4], and are particularly prevalent after fusion surgery (30-46%) [2,5].

The risk of developing chronic pain after surgery has been underestimated in the past [6]. RPP remains widely unrecognized, underdiagnosed, and often inadequately treated. Postoperative pain after spinal fusion is much more severe than in many other surgical procedures [7], and moderate pain is likely to remain even 12 months after surgery [8]. Patients with chronic pain after spinal surgery are often more disabled and report a QOL worse than in other chronic pain conditions [9]. Research on RPP in lumbar degenerative diseases has primarily focused on surgery-related factors such as patient selection, diagnosis, surgical methodology, and postoperative care [2-5]. A previous study on RPP in non-spinal areas identified associations of RPP with depression, psychological susceptibility, stress, and delayed return to work [10]. However, causal relationships were not established due to the cross-sectional nature of each study. In addition, the long-term outcome of patients with RPP was uncertain.

Moreover, few studies have comprehensively addressed physical, psychological, and social factors. Additionally, the definition of RPP varies across studies [2-10]. The Japanese Orthopaedic Association Back Pain Evaluation Questionnaire (JOABPEQ) has a cutoff for validity in the guidelines [11]. In the present study, we defined RPP using the low back pain score of the JOABPEQ.

The purpose of the present study was to examine the impact of RPP up to 24 months after fusion surgery for lumbar degenerative diseases on health-related and disease-specific QOL.

## Materials and methods

Study design

This was a prospective cohort analysis. We conducted this study in compliance with the principles of the Declaration of Helsinki. The research protocol was approved by the Research Ethics Committee of our institution (approval code: 1842). The patients were given the right to opt out of the study.

Patient recruitment

Patients aged ≥ 40 years who had undergone posterior interbody fusion for lumbar degenerative disease between 2012 and 2019 were included in this study. The exclusion criteria were fresh osteoporotic vertebral fractures, history of spinal surgeries, inflammatory conditions, malignancies, cerebrovascular disease, paralysis, primary joint diseases such as active rheumatoid arthritis, moderate-to-severe musculoskeletal disease (e.g., knee, hip, and sacrum), severe psychiatric disorders, and absence of lower-extremity neurological symptoms. Additionally, patients with a JOABPEQ low back pain score of > 80 points were excluded.

Surgical indications were determined by two spine surgeons certified by the Japanese Society for Spine Surgery and Related Research based on the following criteria: (1) intermittent claudication with leg pain and numbness, (2) cauda equina or nerve root compression confirmed on MRI, and (3) ineffectiveness of conservative treatments. Fusion selection criteria included a slip ≥ 5 mm and/or a posterior widening of > 15° on lateral plain X-ray or functional X-ray. All patients underwent posterior lumbar interbody fusion or transforaminal lumbar interbody fusion, accompanied by posterior pedicle screws and local bone in the intervertebral cage. Percutaneous screw placement was performed using an O-arm surgical imaging system and a navigation system (Medtronic, Fridley, MN).

Postoperative protocol

Postoperative rehabilitation was initiated immediately after surgery and was continued until discharge. Patients were weaned from the day after surgery and were allowed to walk with a walker. No braces were used. The length of hospital stay was within 18 days based on the Diagnosis Procedure Combination system. Excessive trunk flexion, extension, and rotation were restricted for three to six months postsurgery. Participation in sports and engagement in heavy labor were permitted after six months postoperatively, before which the patients were required to undergo a physical examination and be cleared by a doctor.

Data collection

Patient characteristics were evaluated according to sex, age, height, weight, and body mass index. Surgery-related factors were evaluated, including the number of fusion levels, operative time, and estimated blood loss. Patient-reported outcome measures were collected preoperatively, as well as at six, 12, and 24 months postoperatively, using the JOABPEQ and SF-36. The JOABPEQ is a validated disease-specific instrument for QOL assessment developed in Japan. It consists of five domains: low back pain, lumbar function, walking ability, social life function, and mental health, which are each scored out of 100 points; the higher the points, the more favorable the results [11].

Statistical analysis

**Figure 1:**
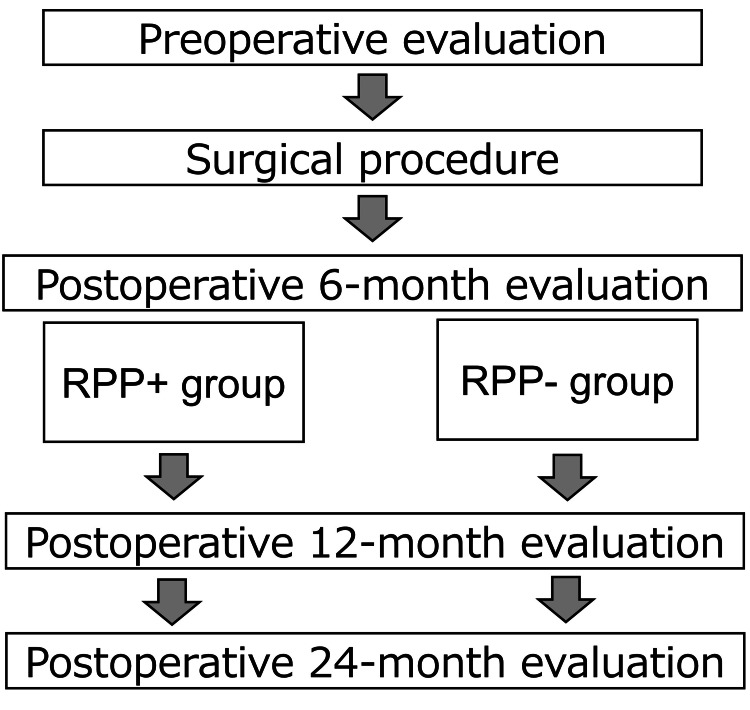
Study design RPP, residual postoperative pain

In the present study, RPP was determined using the low back pain domain in the JOABPEQ. As per an earlier study [11] on the JOABPEQ at six months postoperatively, patients with an improvement of < 20 points compared to the preoperative score were categorized into the RPP+ group, while those with an improvement of ≥ 20 were classified as the RPP- group (Figure 1).

The baseline characteristics were summarized using appropriate statistical methods. The Shapiro-Wilk test was used to assess the normality of the distribution of continuous variables. Parameters between the RPP+ and RPP- groups were compared using t-tests and the Mann-Whitney U test for age, operative time, estimated blood loss, pre- and postoperative JOABPEQ, and SF-36 scores for each subdomain. Categorical variables were presented as absolute numbers and percentages and were analyzed using Fisher’s exact or chi-square tests as deemed appropriate. Longitudinal data were analyzed using repeated measures analysis of variance, followed by a paired t-test with the Shaffer correction. Statistical significance was set at a p-value < 0.05. All statistical analyses were conducted using R Commander (version 4.3.2; R Development Core Team, Vienna, Austria).

## Results

Patient characteristics

**Figure 2:**
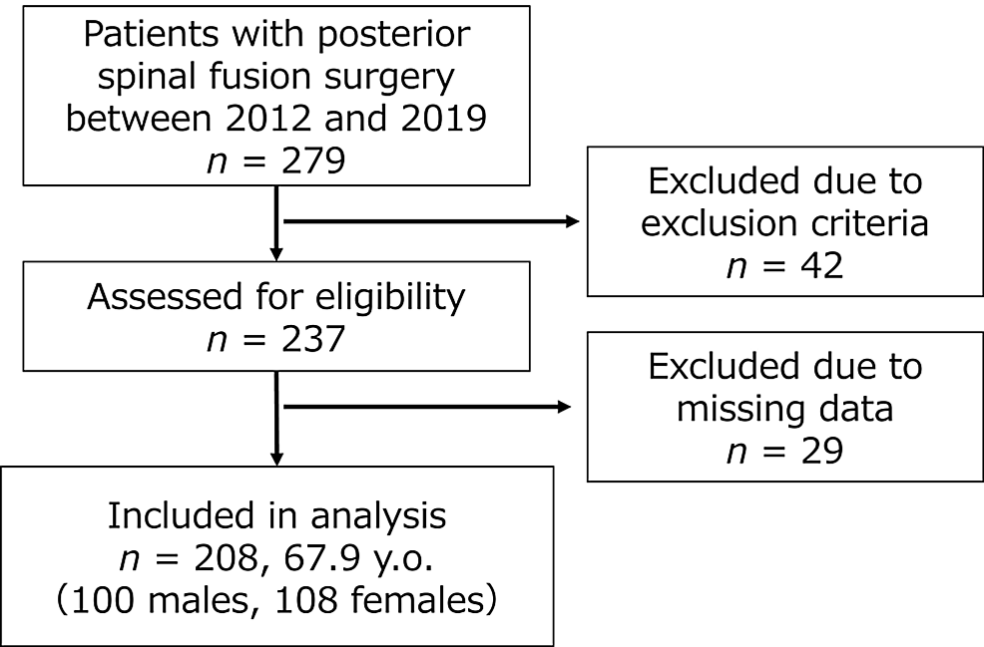
Study flow JOABPEQ, Japanese Orthopaedic Association Back Pain Evaluation Questionnaire; y.o., years old

The study involved 208 participants (100 males and 108 females; mean age 67.9 years, Figure 2). Preoperative diagnoses included degenerative spondylolisthesis (n = 166), foraminal stenosis (n = 31), and isthmic spondylolisthesis (n = 11). Among them, 60 patients belonged to the RPP+ group (27 females; mean age 69.6 ± 8.6 years), while 148 patients were in the RPP- group (81 females; mean age 67.4± 11.4 years). Table 1 displays age, sex, operative time, blood loss, and number of fused vertebral columns. No significant differences in patient characteristics were observed between the RPP+ and RPP- groups.

**Table 1:** Comparison of patient characteristics between the RPP+ and RPP- groups RPP, residual postoperative pain; Op. time, operation time. Data are expressed as mean and SD.

	RPP+ group, n = 60	RPP- group, n = 148	p
Age (y.o)	69.6 ± 8.6	67.4 ± 11.4	0.11
Sex, n (%)			0.09
Female	27 (45.0)	81 (54.7)	
Male	30 (55.0)	67 (45.3)	
Diagnosis, n			0.39
degenerative spondylolisthesis	45	118	
foraminal stenosis	8	21	
isthmic spondylolisthesis	7	9	
Op. time	3:30 ± 1:13	3:17 ± 1:04	0.22
Blood loss (ml)	447.9 ± 269.8	408.6 ± 260.0	0.12
Number of fused vertebral columns			0.43
1	50	122	
2	9	21	
3	1	5	
Smoking, n (%)	7 (11.7)	15 (10.1)	0.35
Length of hospital stay (days)	17.3 ± 2.2	17.2 ± 2.0	0.52

JOABPEQ

**Figure 3:**
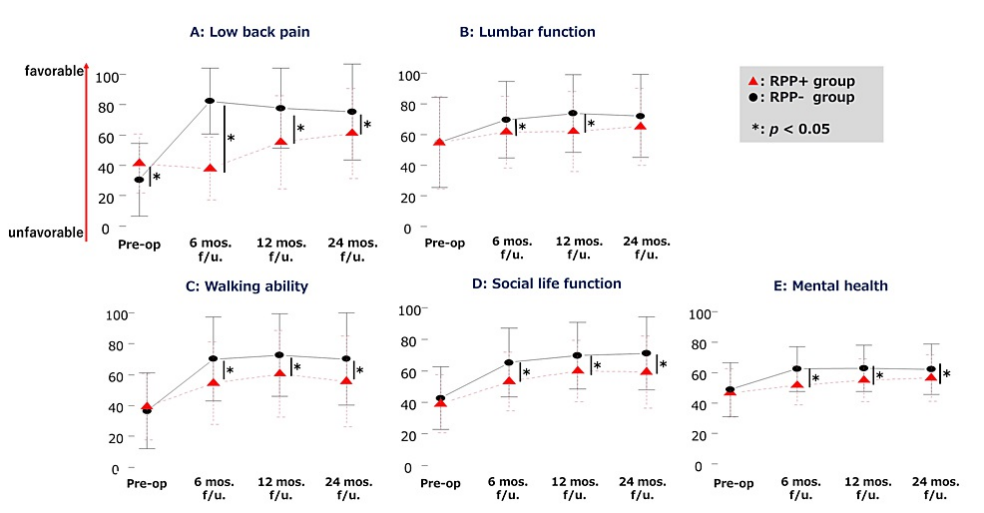
Comparison of JOABPEQ data between the RPP+ and RPP- groups Pre-op., pre-operation; mos., months; f/u, follow-up; RPP, residual postoperative pain * Statistically significant difference

The results of the pre- and postoperative JOABPEQ assessments are shown in Tables 2-3, along with Figure 3. All domains displayed improvement at 24 months postsurgery compared to presurgery in both the groups (p < 0.01). While both groups showed significant improvement in most domains starting from six months postoperatively, the RPP+ group did not exhibit improvement in low back pain and the lumbar function at six months compared to pre-surgery (p = 0.1472; p = 0.3205, respectively). The RPP+ group showed significant improvement in low back pain at 12 months postoperatively (p = 0.0003), but no significant improvement in the lumbar function at 24 months postoperatively (p = 0.0956).

**Table 2:** Pre- and postoperative JOABPEQ scores and SF-36 scores in all patients JOABPEQ, Japanese Orthopaedic Association Back Pain Evaluation Questionnaire; Postop., post-operation; mos., months. Data are expressed as mean and SD. * p < 0.05, vs. pre-op.; ** p < 0.01, vs. pre-op.; † p < 0.05, vs. postop. 6 mos.; †† p < 0.01, vs. postop. 6 mos.; ‡ p < 0.05, vs. postop. 12 mos

	All patients (n = 208)			
Variables	Preoperative	Postop. 6 mos.	Postop. 12 mos.	Postop. 24 mos.
JOABPEQ (0-100)				
Body pain	35.7 ± 23.5	60.0 ± 29.2^**^	66.5 ± 29.3^**, ††^	67.3 ± 32.7^**, ††^
Lumbar spine function	54.7 ± 29.7	65.0 ± 25.1^**^	67.3 ± 26.4^**^	68.2 ± 26.9^**^
Locomotive function	37.8 ± 23.8	61.7 ± 28.2^**^	65.9 ± 28.1^**, †^	61.9 ± 30.6^**, ‡^
Social dysfunction	40.8 ± 20.1	58.8 ± 21.7^**^	64.8 ± 21.2^**, ††^	65.4 ± 23.7^**, ††^
Mentality	48.0 ± 16.7	57.0 ± 14.9^**^	59.4 ± 15.1^**, †^	60.5 ± 15.9^**, ††^
SF-36 (0-100)				
General health	50.1 ± 15.4	54.9 ± 15.5^**^	55.4 ± 17.1^**^	56.1 ± 17.5^**^
Role-emotional	56.2 ± 30.5	67.1 ± 25.8^**^	71.8 ± 25.8^**, †^	74.9 ± 25.0^**, ††^
Role-physical	50.1 ± 26.1	62.5 ± 24.4^**^	67.6 ± 24.1^**, ††^	70.9 ± 24.1^**, ††, ‡^
Body pain	32.4 ± 19.4	60.9 ± 19.9^**^	61.7 ± 22.6^**^	63.7 ± 22.0^**^
Physical function	47.8 ± 21.9	67.2 ± 20.0^**^	68.2 ± 22.3^**^	68.5 ± 22.9^**^
Social functioning	67.2 ± 25.9	77.0 ± 21.5^**^	79.0 ± 22.2^**^	82.1 ± 20.9^**, ††, ‡^
Vitality	49.9 ± 21.2	59.6 ± 18.4^**^	62.1 ± 18.6^**^	64.9 ± 18.4^**, ††, ‡^
Mental health	59.2 ± 20.8	69.9 ± 17.6^**^	72.1 ± 18.6^**^	74.9 ± 16.9^**, ††, ‡^

**Table 3:** Pre- and postoperative Japanese JOABPEQ scores and SF-36 scores in the RPP+ and RPP- groups JOABPEQ, Japanese Orthopaedic Association Back Pain Evaluation Questionnaire; Postop., post-operation; mos., months; RPP, residual postoperative pain. Data are expressed as mean and SD. * p < 0.05, vs. pre-op.; ** p < 0.01, vs. pre-op.; † p < 0.05, vs. postop. 6 mos.; †† p < 0.01, vs. postop. 6 mos

	Preoperative		Postop. 6 mos.		Postop. 12 mos.		Postop. 24 mos.	
Variables	RPP+ group n ＝ 60	RPP- group n = 148	RPP+ group	RPP- group	RPP+ group	RPP- group	RPP+ group	RPP- group
JOABPEQ (0-100)								
Low back pain	41.4 ± 19.7	29.9 ± 24.1	38.1 ± 19.6	81.7 ± 22.1^**^	56.2 ± 30.6^**, ††^	76.8 ± 26.6^**, †^	60.1 ± 30.4^**, ††^	74.4 ± 32.9^**, ††^
Lumbar function	54.4 ± 30.9	54.9 ± 29.3	60.6 ± 23.0	69.4 ± 25.6^**^	61.2 ± 26.0	73.4 ± 25.8^**^	64.2 ± 24.9	72.2 ± 27.5^**^
Walking ability	39.3 ± 21.6	36.3 ± 24.6	54.2 ± 27.2^**^	69.2 ± 27.6^**^	59.8 ± 28.1^**^	72.0 ± 27.4^**^	54.7 ± 29.2^**^	69.1 ± 30.3^**^
Social life function	39.0 ± 18.9	42.7 ± 20.6	53.0 ± 18.9^**^	64.7 ± 22.0^**^	60.0 ± 19.6^**, †^	69.6 ± 21.3^**, ††^	59.6 ± 22.4^**, †^	71.1 ± 23.5^**, ††^
Mental health	47.3 ± 15.3	48.7 ± 17.2	51.6 ± 12.7^**^	62.3 ± 14.6^**^	55.8 ± 13.5^**, †^	63.0 ± 15.3^**^	57.8 ± 14.2^**, ††^	63.3 ± 16.3^**^
SF-36 (0-100)								
General health	47.9 ± 14.7	53.7 ± 15.4	49.5 ± 12.5	60.4 ± 15.5^**^	50.7 ± 13.5	60.1 ± 17.6^**^	53.0 ± 14.6	59.3 ± 18.3^**^
Role-emotional	56.7 ± 27.4	55.8 ± 31.8	59.9 ± 24.1	74.4 ± 25.3^**^	64.9 ± 27.8	78.7 ± 24.0^**^	70.4 ± 27.6^*, †^	79.4 ± 23.4^**^
Role-physical	52.8 ± 21.5	47.5 ± 27.7	55.8 ± 22.1	69.3 ± 24.3^**^	62.0 ± 22.2^*, †^	73.3 ± 24.2^**, †^	65.6 ± 23.8^**, ††^	76.2 ± 23.6^**, ††^
Body pain	30.2 ± 18.1	33.4 ± 19.8	49.0 ± 16.9^**^	65.7 ± 19.0^**^	53.3 ± 19.5^**^	65.1 ± 22.9^**^	55.4 ± 19.7^**, ††^	67.0 ± 22.0^**^
Physical function	49.3 ± 22.2	46.4 ± 21.8	62.4 ± 18.4^**^	71.9 ± 20.0^**^	64.8 ± 21.8^**^	71.6 ± 22.3^**^	64.2 ± 22.9^**^	72.9 ± 22.5^**^
Social functioning	68.1 ± 26.9	66.2 ± 25.5	70.6 ± 22.0	83.4 ± 20.3^**^	74.0 ± 24.9	84.1 ± 20.3^**^	78.3 ± 21.9^*, †^	85.8 ± 20.1^**^
Vitality	50.6 ± 19.7	49.2 ± 21.8	52.9 ± 18.5	66.3 ± 16.9^**^	58.0 ± 18.9^*^	66.2 ± 18.0^**^	62.0 ± 17.8^**, ††^	67.8 ± 18.5^**^
Mental health	59.8 ± 19.3	58.5 ± 21.4	63.3 ± 18.7	76.5 ± 15.6^**^	68.3 ± 19.3^**, †^	75.8 ± 17.9^**^	72.3 ± 16.5^**, ††^	77.4 ± 16.9^**^

In inter-group comparisons, the RPP+ group showed lower low back pain scores presurgery compared to the RPP- group (p = 0.0013). Preoperative scores for lumbar function, walking ability, social life function, and mental health did not significantly differ between the RPP+ and RPP- groups. Nonetheless, the lumbar function in the RPP+ group was notably lower than in the RPP- group at six and 12 months postsurgery (p = 0.0210, p = 0.0023, respectively). Walking ability, social life function, and mental health were each notably lower in the RPP+ group than in the RPP- group at six, 12, and 24 months postoperatively (p = 0.0020, p = 0.0013, p = 0.0243, respectively). In domains other than low back pain within the RPP+ group, most improvement (89-100%) that occurred during 24-month postoperative period had been achieved within the first six months.

SF-36

**Figure 4:**
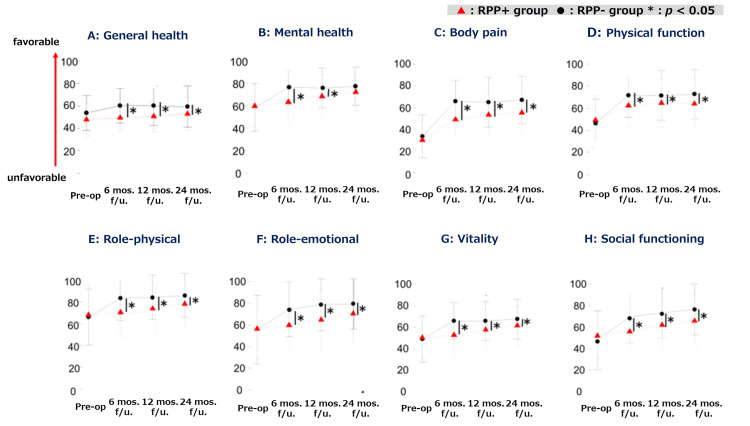
Comparison of SF-36 data between the RPP+ and RPP- groups Pre-op., preoperation; mos., months; f/u, follow-up; RPP, residual postoperative pain * Statistically significant difference

The pre- and postoperative SF-36 assessment results are shown in Tables 2-3 and Figure 4. All domains showed improvement at 24 months postoperatively compared to the preoperative scores in both groups (p < 0.001). At 24 months postoperatively, the RPP+ group demonstrated a significant decrease compared to the RPP- group in all subdomains, excluding mental health (Table 2). The RPP+ group showed no significant improvement in general health at 24 months (p = 0.1011). On the other hand, improvements were observed in pole-physical, vitality, and mental health at 12 months postoperatively (p = 0.0297, p = 0.0331, p = 0.0032, respectively), as well as in role-emotional and social functioning at 24 months postoperatively. The majority of improvements (85-100%) observed over the 24-month postoperative period occurred within the first six months.

## Discussion

The current study investigated the impact of residual pain after posterior fusion surgery for lumbar degenerative disease over 24 months postoperatively. The results indicated that residual pain at six months postoperatively was an important predictive factor affecting QOL improvement up to 24 months postoperatively. Its impact extended to functional, social, and psychological aspects. Notably, the influence on the lumbar function of the JOABPEQ and the general health domains of the SF-36 was significant, with a discernible lag in improvement observed in role-emotional, role-physical, vitality, social functioning, and mental health. To the best of our knowledge, this is the first study to demonstrate the effect of RPP on QOL outcomes after posterior fusion surgery for lumbar degenerative disease using the JOABPEQ and SF-36. The prevalence of RPP in this study was 28.4%, which is similar to aligns with or is slightly lower than the reported rates of RPP after lumbar fusion surgery, ranging from 30% to 46%, in previous studies. The pain scores of both the JOABPEQ and SF-36 in the RPP+ group demonstrated improvement throughout the postoperative follow-up. However, these scores remained significantly lower than those observed in the RPP- group at 24 months postoperatively, indicating prolonged pain.

Impact of residual pain

In the current investigation, all domains of the JOABPEQ exhibited enhancement at 24 months postoperatively compared with the preoperative baseline in both cohorts. Additionally, most domains showed significant improvement starting at six months postoperatively. Low back pain and lumbar function in the RPP+ group exhibited no improvement at six months postoperatively compared to the preoperative assessment. Nevertheless, low back pain demonstrated a significant improvement at 12 months postoperatively, whereas lumbar function did not show significant improvement at 24 months postoperatively, compared to the preoperative assessment.

The questions on lumbar function in the JOABPEQ encompass activities of daily living involving trunk flexion and rotation. After fusion surgery, lumbar spine motion restrictions continue for three to six months until bony fusion is complete, and in some cases, a rigid brace is used [12]. In the current study, postoperative bracing was not employed; however, heavy lifting and excessive trunk movements were restricted for three to six months after surgery. The RPP+ group may have experienced prolonged functional limitations attributed to reduced lumbar spinal flexibility resulting from pain. Conversely, prolonged periods of inactivity pose a risk for chronic pain [13], and the RPP+ group may have experienced persistent pain due to the avoidance of lumbar spine motion. In contrast, fusion surgery is known to induce lumbar spine stiffness, affecting the activities of daily living. Fusion involving three or more vertebral columns has been reported to exert a robust effect on stiffness [14]; however, in the present study, the patients generally underwent one- or two-level fusion, with no discernible difference observed between the RPP+ and RPP- groups in the number of fused vertebral columns.

Surgery for lumbar spinal stenosis has been reported to lead to a significant reduction in pain and functional impairment within the first three months postoperatively, with little improvement from six months until five years after surgery [15]. While the previous study included decompression without fusion procedures, the present study exclusively included posterior fusion surgeries. As a result, the majority of improvements observed during the 24-month postoperative period occurred within the first six months (RPP+ group, 85-98%; RPP- group, 90-100%).

In the RPP- group, all domains were improved at six months postoperatively compared to the preoperatively assessment, whereas only physical impairment was improved at the same time point in the RPP+ group. Prolonged impairment related to mobility, social participation, and physical activity may have had an impact on the health of the patients in the RPP+ group.

Strengths and limitations

The present study has several strengths. First, to the best of our knowledge, this is the first study to demonstrate the impact of residual pain on QOL outcomes after posterior fusion surgery for lumbar degenerative disease using the JOABPEQ and SF-36. Our findings indicate that residual pain at six months postoperatively affects QOL improvement for up to 24 months. Second, a detailed examination of the subdomains revealed that the impact of RPP extended to functional, social, and psychological aspects. Low back pain in the preoperative JOABPEQ was lower in the RPP+ group than in the RPP- group, indicating that it may be an important predictor of the occurrence of RPP.

Third, our investigation showed that the changes in scores for the JOABPEQ and SF-36 varied across different subdomains. In particular, distinctive changes were observed in the lumbar function of the JOABPEQ in the RPP+ group. These results suggest that lumbar dysfunction after fusion surgery may be an important factor related to RPP. The results also showed a significant impact on general health of the SF-36 in the RPP+ group. Furthermore, our investigation revealed delays in the improvement of performing daily activities, socialization, and vitality, suggesting potential delay in postoperative socialization, and returning to domestic roles. Identifying subdomain scores at each time point will allow the evaluation of treatment efficacy and points for rehabilitation and other interventions. In addition to improving pain and function, an early postoperative rehabilitation program may be required to promote social participation and improve physical activity.

Despite the strengths of our study, it has several limitations. First, because this was a single-center prospective study, a multicenter prospective study may be required for generalizability of the results. In the present study, the incidence of RPP and the age demographics of the patients align closely with those of prior studies [5-8]. Out of the 237 patients who underwent surgery during the current study period, with the exclusion of those with mild preoperative pain, 208 completed the final 24-month follow-up (follow-up rate: 88%).

Second, the etiology of RPP remains unclear. While chronic pain and postoperative clinical outcomes have been associated with physical function and psychosocial factors [16,17], there were no significant differences in social or psychological impairment and physical function between the preoperative RPP+ and RPP- groups in the present study. Preoperative low back pain score of the JOABPEQ was significantly higher in the RPP+ group, suggesting that patients' expectations regarding surgery may have influenced their residual symptoms. Furthermore, details of psychosocial factors (e.g., the presence of workers' compensation insurance, educational and income levels, and the presence of pain catastrophizing) were not examined, leaving open the possibility of their impact on the results.

Third, the changes in residual pain from the immediate postoperative phase to six months were unknown. The identification of postoperative chronic pain may be feasible at an earlier stage [18]. In the present study, the patients underwent early rehabilitation by a physical therapist after lumbar spine surgery. Early improvement in gait and mobility is reported to contribute to the prevention of postoperative complications and the improvement of long-term outcomes [19]. All patients underwent rehabilitation for one hour a day, five times a week, for approximately two weeks from the immediate postoperative period to the time of discharge. However, the duration and level of postoperative physical activity are unknown. Prospective studies focusing on the relationship between postoperative physical activity and RPP using activity trackers are required to clarify these factors.

## Conclusions

The present study used the JOABPEQ and SF-36 to investigate the impact of residual pain after posterior fusion surgery on QOL outcomes. The study revealed that RPP at six months postoperatively affects QOL improvement for up to 24 months. The impact of RPP extended to functional, social, and psychological aspects. In particular, the lumbar function in the JOABPEQ showed a specific process in the RPP+ group. These results suggest that lumbar dysfunction after fusion surgery may be an important factor related to RPP.

The SF-36 process differed by subdomain. The presence of residual pain at six months postoperatively may indicate that general health is unlikely to improve and that role-emotional, role-physical, vitality, social functioning, and mental health may show delayed progression.

## References

[1] Ishimoto Y, Yoshimura N, Muraki S (2012). Prevalence of symptomatic lumbar spinal stenosis and its association with physical performance in a population-based cohort in Japan: the Wakayama Spine Study. Osteoarthritis Cartilage.

[2] Chan CW, Peng P (2011). Failed back surgery syndrome. Pain Med.

[3] Sebaaly A, Lahoud MJ, Rizkallah M, Kreichati G, Kharrat K (2018). Etiology, evaluation, and treatment of failed back surgery syndrome. Asian Spine J.

[4] Inoue S, Kamiya M, Nishihara M, Arai YP, Ikemoto T, Ushida T (2017). Prevalence, characteristics, and burden of failed back surgery syndrome: the influence of various residual symptoms on patient satisfaction and quality of life as assessed by a nationwide Internet survey in Japan. J Pain Res.

[5] Shapiro CM (2014). The failed back surgery syndrome: pitfalls surrounding evaluation and treatment. Phys Med Rehabil Clin N Am.

[6] Skolasky RL, Wegener ST, Maggard AM, Riley LH 3rd (2014). The impact of reduction of pain after lumbar spine surgery: the relationship between changes in pain and physical function and disability. Spine (Phila Pa 1976).

[7] Gerbershagen HJ, Aduckathil S, van Wijck AJ, Peelen LM, Kalkman CJ, Meissner W (2013). Pain intensity on the first day after surgery: a prospective cohort study comparing 179 surgical procedures. Anesthesiology.

[8] Fletcher D, Stamer UM, Pogatzki-Zahn E (2015). Chronic postsurgical pain in Europe: an observational study. Eur J Anaesthesiol.

[9] Manca A, Eldabe S, Buchser E, Kumar K, Taylor RS (2010). Relationship between health-related quality of life, pain, and functional disability in neuropathic pain patients with failed back surgery syndrome. Value Health.

[10] Hinrichs-Rocker A, Schulz K, Järvinen I, Lefering R, Simanski C, Neugebauer EA (2009). Psychosocial predictors and correlates for chronic post-surgical pain (CPSP) - a systematic review. Eur J Pain.

[11] Fukui M, Chiba K, Kawakami M (2009). JOA Back Pain Evaluation Questionnaire (JOABPEQ)/JOA Cervical Myelopathy Evaluation Questionnaire (JOACMEQ). The report on the development of revised versions. April 16, 2007. The Subcommittee of the Clinical Outcome Committee of the Japanese Orthopaedic Association on Low Back Pain and Cervical Myelopathy Evaluation. J Orthop Sci.

[12] van Erp RM, Jelsma J, Huijnen IP, Lundberg M, Willems PC, Smeets RJ (2018). Spinal surgeons’ opinions on pre- and postoperative rehabilitation in patients undergoing lumbar spinal fusion surgery: a survey-based study in the Netherlands and Sweden. Spine (Phila Pa 1976).

[13] Qaseem A, Wilt TJ, McLean RM (2017). Noninvasive treatments for acute, subacute, and chronic low back pain: a clinical practice guideline from the American College of Physicians. Ann Intern Med.

[14] Kimura H, Fujibayashi S, Otsuki B, Takahashi Y, Nakayama T, Matsuda S (2016). Effects of lumbar stiffness after lumbar fusion surgery on activities of daily living. Spine (Phila Pa 1976).

[15] Fritsch CG, Ferreira ML, Maher CG, Herbert RD, Pinto RZ, Koes B, Ferreira PH (2017). The clinical course of pain and disability following surgery for spinal stenosis: a systematic review and meta-analysis of cohort studies. Eur Spine J.

[16] Kamper SJ, Apeldoorn AT, Chiarotto A, Smeets RJ, Ostelo RW, Guzman J, van Tulder MW (2014). Multidisciplinary biopsychosocial rehabilitation for chronic low back pain. Cochrane Database Syst Rev.

[17] Sugimoto S, Nagai S, Ito K (2024). The impact of frailty on surgical outcome of patients with lumbar spinal canal stenosis. Spine Surg Relat Res.

[18] Macrae WA (2008). Chronic post-surgical pain: 10 years on. Br J Anaesth.

[19] Kalisch BJ, Lee S, Dabney BW (2014). Outcomes of inpatient mobilization: a literature review. J Clin Nurs.

